# Study on Fatigue Coefficient of Airline Pilots

**DOI:** 10.3389/fpsyg.2022.865342

**Published:** 2022-05-11

**Authors:** Peiwen Zhang, Wenke Zhao, Lan Shi, Yu Wang, Hong Sun, Zhen Sun

**Affiliations:** ^1^School of Economics and Management, Civil Aviation Flight University of China, Guanghan, China; ^2^School of Statistics, Southwest University of Finance and Economics, Chengdu, China; ^3^College of Foreign Languages, Civil Aviation Flight University of China, Guanghan, China; ^4^Scientific Research Base, Civil Aviation Flight University of China, Guanghan, China

**Keywords:** airline pilots, fatigue feeling, questionnaire, Kaiser–Meyer–Olkin, Bartlett’s Test of Sphericity, analysis of variance

## Abstract

This paper uses the Multidimensional Fatigue Inventory (MFI-16) to investigate the fatigue status of pilots, and the reliability and validity of the scale are tested by Cronbach’s α and exploratory factor analysis. The founding shows that mild fatigue and above accounted for 67.7%. For further quantify the impact of different flights on pilots’ fatigue, research improves the fatigue coefficient model based on the results of pilot fatigue feeling questionnaire. Combined with multifactor analysis of variance and multiple linear regression, it is found that the independent variables have different and positive effects on the dependent variables, and there is no multicollinearity. Through the actual test, its accuracy is improved by 16.7% compared with the original model.

## Introduction

The continuous expansion of civil aviation industry leads to the increasing workload of existing pilots. During periods of routine flights, Unreasonable duty time starting earlier or ending later affects the state of mind (Caldwell, 2005). Meanwhile, the frequent operation of takeoff and landing leads to a great workload during short-haul flights. All of these induce fatigue accompanied by the decline of decision-making power, attention, and reflection speed (Maarten et al., 2005). Research found that the proportion of participants classified as severe fatigue reached 75%, respectively, (Jackson and Earl, 2006). In view of this, fatigue has gradually become a key factor threatening flight safety. Thus, effective fatigue monitoring and warning in time are of great significance to ensure flight safety.

Combined with online questionnaire and risk factors provided by the airlines, it is found that pilots whose circadian rhythm is eveningness have a higher risk of fatigue (Van Drongelen et al., 2017). When the duty time is completely at the trough of circadian rhythm, the probability of serious fatigue of pilots is the highest (Sallinen et al., 2020). Fatigue may be unavoidable due to the nature of the pilots’ job. It is noteworthy that the civil aviation industry gradually began to take corresponding countermeasures against fatigue in order to improve operation efficiency and safety (Akerstedt, 2000). For example, proper rest in designated rest facilities or flight deck can help reduce the impact of fatigue on mobility and alertness (Hartzler, 2014). Meanwhile, the fatigue bio mathematical model can improve the fatigue caused by unreasonable scheduling (Cabon et al., 2012). However, fatigue assessment is an important part of fatigue management. The fatigue assessment based on brain dynamics has been widely used in the field of fatigue detection at present. It mainly gives the assessment through pilots’ physiological feedback information such as long-time eye closure, yawning, blood pressure and electrocardiogram (Lal and Craig, 2001; Lal et al., 2003; Azarnoosh et al., 2011; Craig et al., 2012). Since the pilots’ actions or facial expressions are recorded, this kind of evaluation method may have privacy problems. Therefore, the evaluation of pilots’ fatigue mainly depends on personal subjective feedback, that is, inferring workload or brain fatigue status through subjective feelings, including Karolinska Sleepiness Scale (KSS; Kaida et al., 2006).

Since 2011, the Civil Aviation Administration of China (CAAC) has been measuring the fatigue coefficients based on the total flight hours and the number of available crews per month to monitor the workload level of each pilot. However, the existing fatigue coefficient models only consider the total flight time, do not take into account the impact of different types of flight tasks on fatigue perception. While the models only reflect the overall fatigue status, and are not suitable for monitoring the fatigue of individual pilots. In this regard, according to the working characteristics of pilots, this study issued survey scales to pilots for collecting their subjective fatigue data, in order to establish pilot fatigue coefficient monitoring model.

## Design and Implementation of Questionnaire

The investigation is divided into two stages: the investigation of the overall fatigue status of pilots, and pilots’ fatigue perception of different flight types. It is of great significance to carry out a follow-up investigation if the current situation of pilot fatigue is widespread. Therefore, pilot fatigue scale questionnaire improved on the basis of MFI-20 scale (Smets et al., 1996) and pilot fatigue feeling questionnaire are designed. All questionnaires adopt the principle of unified design: reasonable levels, well-defined content with no objection, moderate length, neutral problem with no induction, and convenient for data analysis. The questionnaire includes: cover and instructions, personal information, and questions and options.

The respondents are front-line pilots under the jurisdiction of CAAC Southwest Regional Administration. The questionnaires were distributed online, which can reduce the project cost and improve the work efficiency. At the same time, it can enable the pilots to complete the questionnaires more accurately. The distribution amount of fatigue scale questionnaires is determined according to 10 times of the item, a total of 150 copies, and 133 copies are effectively recovered as the overall sample of the fatigue status survey, as shown in Table 1. It is found that mild fatigue and above accounted for 67.7%, which is universal. Thus, pilot fatigue feeling questionnaire takes the front-line pilots under the jurisdiction of CAAC Southwest Regional Administration as the survey population, in order to better represent the overall characteristics. The questionnaire type is non scale, so the sample size is 700, and 599 copies effectively recovered. All questionnaires adopt the principle of unified screening: missing answers to questionnaire items, contradiction between positive and negative questions, the answers to the overall options are regular or consistent, failure to answer as instructed, multiple-choice answers for questions, and repeat the answer.

**TABLE 1:** Basic personal information statistics of airline pilots.

Project	Category	Number	Proportion	Project	Category	Number	Proportion
Age	<25	6	4.5%	Marital Status	Unmarried	21	15.8%
	26–30	33	24.8%		Married	109	82.0%
	31–35	67	50.4%		Other	3	2.3%
	36–40	22	16.5%	Aircraft type	Narrow-body	111	83.5%
	41–45	4	3.0%		Wide-body	22	16.5%
	46–50	1	0.8%		Other	0	0
	>50	0	0	Location	Air China	47	35.3%
Flight years	1–5	44	33.1%		West Air	75	56.4%
	6–10	51	38.3%		Other	11	8.3%
	11–15	29	21.8%	Frequency of physical exercise	Three or more times a week	38	28.6%
	>16	9	6.8%		Less than 3 times a week	90	67.7%
Total flight hours (h)	0–5,000	65	48.9%		No	5	3.8%
	5,001–10,000	38	28.6%	Physical Condition	Well	8	6.0%
	10,001–15,000	22	16.5%		Better	87	65.4%
	15,001–20,000	5	3.8%		General	38	28.6%
	>20,000	3	2.3%		Poor	0	0
Technical level	Co-pilot	69	51.9%				
	Captain	33	24.8%				
	Instructor	31	23.3%				
	Other	0	0				

*The physical condition of the pilot is judged according to the medical report in the regular physical examination. Among them, well indicates that there is no abnormality in the body, better indicates that there are some problems in the body but do not affect the flight, general indicates that the body needs to be recuperated until it meets the flight standard, and poor indicates that it is unable to undertake subsequent flight missions.

## Pilot Fatigue Status

### Reliability Test

The reliability test uses Cronbach’s α, which is usually required to be greater than 0.7 (Adamson and Prion, 2013). The total Cronbach’s α of the scale was 0.959, and the Cronbach’s α of the four dimensions of overall fatigue, reduced power, physical fatigue, and mental fatigue were 0.895, 0.919, 0.892, and 0.909. These indicate that the result of this survey has a higher reliability.

### Validity Test

To verify whether it is appropriate to use a question to represent a variable, exploratory factor analysis was used to test the rationality of the structure of the MFI-16 scale. The main terms related to exploratory factor analysis are Kaiser–Meyer–Olkin (KMO; Karen et al., 2013) and Bartlett’s Test of Sphericity (Cleophas and Zwinderman, 2012). As the result of the KMO and Bartlett test (0.937), factor analysis was administered to the survey, which can be seen from Table 2.

**TABLE 2:** Kaiser–Meyer–Olkin and Bartlett’s Test of Sphericity.

Kaiser–Meyer–Olkin measure of sampling adequacy		0.937
Bartlett’s Test of Sphericity	Approx of Chi-Square	1886.881
	df	120
	sig	0.000

In Table 3, the values of extract column are more than 0.5, and some are close to or more than 0.8. It shows that the factors can extract the information of each question very well, so all of 16 entries are retained.

**TABLE 3:** The common factor variance of 16 questions.

Question	Initial	Extract
1. I feel very well in spirit	1.000	0.836
2. I feel tired and low energy	1.000	0.814
3. I want to do all kinds of things I like	1.000	0.843
4. I can take on a lot of work	1.000	0.690
5. I can concentrate well	1.000	0.730
6. I don’t want to do anything	1.000	0.812
7. I don’t really want to move, I just want to go rest	1.000	0.811
8. I forget things easily, memory loss	1.000	0.822
9. I have a hard time concentrating	1.000	0.852
10. I feel energized	1.000	0.809
11. I have a lot of things I want to do	1.000	0.809
12. I feel in very good physical condition	1.000	0.827
13. I have a good memory	1.000	0.788
14. I get tired easily	1.000	0.704
15. I don’t feel well	1.000	0.779
16. I think only a small amount of work can be done	1.000	0.775

*Extraction method: principal component analysis.

It can be seen that the eigenvalues of factors are greater than 0.8, the cumulative variance contribution rate of factors is 79.387%. while the variance contribution rate of each factor after rotation is above 18%, which indicates that the results of factor analysis are good, as can be seen from Table 4.

**TABLE 4:** Total variance explained by the 4 factors.

Factor	Initial eigenvalue			Rotation sum of squares loading		
	Eigenvalues	Variance ratio %	Cumulative %	Eigenvalues	Variance ratio %	Cumulative %
1	10.010	62.564	62.564	3.396	21.227	21.227
2	0.990	6.186	68.750	3.238	20.236	41.462
3	0.868	5.425	74.175	3.183	19.893	61.355
4	0.834	5.212	79.387	2.885	18.032	79.387

After confirming the number of exploration factors, it is also necessary to look at the correspondence between the factors and the questions. If a question has a relatively high value of the factor loading coefficient corresponding to a factor, it means that the question has a relatively high correlation with that factor, and then the question should be attributed to that factor. It was found that the factor loading coefficients of the corresponding questions were all greater than 0.4, indicating the generated correspondence between the factors and the topics showed consistency with expectations, as shown in Table 5. Overall fatigue includes T1 (referring to topic 1, and so on later), T2, T10, and T14. Physical fatigue includes T4, T12, T15, and T16. Mental fatigue includes T5, T8, T9, and T13. Power reduction includes T3, T6, T7, and T11.

**TABLE 5:** Component matrix after maximum variance rotation for 16 topics.

Question	Factor			
	1	2	3	4
1. I feel very well in spirit				0.808
2. I feel tired and low energy				0.789
10. I feel energized				0.657
14. I get tired easily				0.553
3. I want to do all kinds of things I like			0.830	
6. I don’t want to do anything			0.719	
7. I don’t really want to move, I just want to go rest			0.666	
11. I have a lot of things I want to do			0.721	
4. I can take on a lot of work		0.755		
12. I feel in very good physical condition		0.734		
15. I don’t feel well		0.703		
16. I think only a small amount of work can be done		0.677		
5. I can concentrate well	0.625			
8. I forget things easily, memory loss	0.778			
9. I have a hard time concentrating	0.816			
13. I have a good memory	0.748			

### Statistical Analysis of Fatigue Scales

According to the five-point Likert scale, several sentences describing variables are drawn up, and the attitude expressed in the sentences tends to be positive and negative, so as to carry out straightforward and reverse scored (Weber et al., 2014). The scale compiled in this paper contains 4 dimensions, including overall fatigue, physical fatigue, reduced power, and mental fatigue. “Overall fatigue” is a general description of a person’s state, “Physical fatigue” is to examine the feeling of physical state, “Reduced motivation” is to examine the change in motivation to do things, and “Mental fatigue” is an examination of mental changes. Each dimension consists of 4 items, and a total of 16 items. The theoretical minimum value of the total score is 16 points and the maximum value is 80 points. Combined with expert opinions and scoring rules, the degree of fatigue could be classified as no fatigue (16–35 points), mild fatigue (36–55 points), and severe fatigue (56–80 points), as shown in Table 6.

**TABLE 6:** Correspondence table between MFI-16 scale scores and 3 types of fatigue levels.

Score	16–35	26–55	56–80
Degree	No fatigue	Mild fatigue	Severe fatigue

The distribution of fatigue levels among airline pilots was obtained after the statistics: 32.3% of pilots were not fatigued, 63.9% were mildly fatigued, and 3.8% were severely fatigued. The results show that the phenomenon of flight fatigue does exist and is relatively common.

## Fatigue Coefficient Model Design

### Improvement Ideas

The existing pilot fatigue coefficient model only reflects the overall fatigue status, and the investigation angle is relatively single. Work starting earlier or ending later tends to disrupt the work and rest routine, resulting in pilots flying in a poor mental state. Therefore, we mainly focus on the duty time and whether it involves plateau airports when improving the fatigue coefficient model. In theory, a preliminary analysis indicated that there were some decrements in alertness, reductions in sleep, and disruptions of other personal activities within 12 working hours (Rosa et al., 1989). Meanwhile, near 22:00, all test groups showed weak vigilance (Milia et al., 2005). Thus, the flights are divided into morning flights (duty period is before 08:00), daytime flights (duty period is between 08:00 and 22:00) and evening flights (duty period is after 22:00). Flights often involve varied topographies, such as plateau area. Due to the rapid change of wind speed and direction, turbulence and wind shear are more frequent than plain area. Therefore, pilots require increased effort to keep the plane steady. The regulations on the operation and management of air carrier plateau airport issued by the CAAC point out that airports with an altitude of 1,500 meters and above are plateau aircraft. Thus, flights are divided into plateau flights (the altitude of the airport is more than 1,500 meters) and plain flights (the altitude of the airport is lower than 1,500 meters). In view of this, the total monthly flight time is split into morning-plain flight, daytime-plain flight, evening-plain flight, morning-plateau flight, daytime-plateau flight, and evening-plateau flight. In practice, the Civil Aviation Administration of China and airlines also usually use this classification method.

By weighting the flight time of different types of flights to correct the monthly time-of-flight, we achieve a preliminary target model. The improved model can take into account the differentiated fatigue feelings brought by flying different types of flights, and it is closer to the real feelings. The improved fatigue coefficient model is as follows.


(1) $$F=\frac{T_{1}\times a_{1}+T_{2}\times a_{2}+T_{3}\times a_{3}+T_{4}\times a_{4}+T_{5}\times a_{5}+T_{6}}{A}$$

where *F* is the pilots’ fatigue coefficient, *T*_1_ is the time-of-flight of morning-plain shift per month, *T*_2_ is the time-of-flight of evening-plain shift per month, *T*_3_ is the time-of-flight of morning-plateau shift per month, *T*_4_ is the time-of-flight of evening-plateau shift per month, *T*_5_ is the time-of-flight of daytime-plateau shift per month, and *T*_6_ is the time-of-flight of daytime-plain shift per month. *α_1_* to *α_5_* are the fatigue correction coefficient of factors. *A* is the critical fatigue flight hours of daytime-plain shift per month.

### Improvement Process

Since the relationship between categorical and quantitative data is studied, analysis of variance (ANOVA) is used. It can usually be divided into one-way analysis of variance and multi-way analysis of variance.

When processing the data, the text is converted into numbers. For flights in different duty periods, 0 represents “daytime flight,” 1 represents “evening flight,” and 2 represents “morning flight.” For whether it is a plateau flight, 0 represents “plain flight,” and 1 represents “plateau flight,” as shown in Table 7.

**TABLE 7:** Multi-factor ANOVA between-subjects factor.

		Label	Sample size
Flights in different duty periods	0	Daytime flight	599
	1	Evening flight	1,198
	2	Morning flight	1,198
Whether it is a plateau flight	0	Plain flight	1,198
	1	Plateau flight	1,797

It can be seen from Table 8 that the “Flights in different duty periods,” “whether it is a plateau flight,” and “Flights in different duty periods * whether it is a plateau flight” are significant and have different effects on fatigue feeling (*p* < 0.05).

**TABLE 8:** Multi-factor ANOVA between-subjects effect test.

Source	Type III SS	df	σ	*F*	*p*
Modified model	1410.763	4	352.691	92.414	0.000
Intercept	41338.440	1	41338.440	10831.679	0.000
Flights in different duty periods	1097.647	2	548.823	143.805	0.000
Whether it is a plateau flight	842.755	1	842.755	220.822	0.000
Flights in different duty periods* Whether it is a plateau flight	17.199	1	17.199	4.507	0.034
Error	11411.152	2,990	3.816		
Total	60287.000	2,995			
Corrected total	12821.915	2,994			

In order to study the difference of fatigue feeling between pilots flying flights in different duty periods and plateau flights, a multiple regression model is constructed, as shown in formula (2), where *y* is the fatigue feeling of pilots, *X*_1_ is the morning flight, *X*_2_ is the evening flight, and *X*_3_ is the plateau flight. The regression model is fitted with the sample data, results indicate that these independent variables will have an impact on the dependent variable (*P* = 0.000), and they will have a significant positive impact on the dependent variable (*B* > 0). Meanwhile, there is no multicollinearity problem (*VIF* < 5), as shown in Table 9.


(2) $$y=\alpha_{1}*X_{1}+\alpha_{2}*X_{2}+\alpha_{3}*X_{3}+\beta$$

**TABLE 9:** Table of linear regression coefficients.

Model		Unstandardized coefficients		Standardized coefficients	*t*	*p*	Collinearity statistics	
		*B*	Standard error	Beta			Tolerance	VIF
1	β	1.196	0.011		105.909	0.000		
	α_1_	0.175	0.011	0.414	16.555	0.000	0.476	2.100
	α_2_	0.152	0.011	0.359	14.367	0.000	0.476	2.100
	α_3_	0.119	0.008	0.281	14.851	0.000	0.833	1.200

It can be seen from Table 9 that the fatigue feeling of morning flight is 0.175 higher than that of daytime flight on average, the evening-plain flight is 0.152 higher than that of the daytime-plain flight on average, the daytime-plateau flight is 0.119 higher than that of daytime-plain flight on average. The above analysis shows that the fatigue feeling of morning flight is greater than that of evening flight, that of evening flight is greater than that of daytime flight, and that of plateau flight is greater than that of plain flight. Further, we can calculate the fatigue feeling value of different types of flights. The fatigue feeling value of morning plain flight is 1.371, evening-plain flight is 1.348, morning-plateau flight is 1.490, evening-plateau flight is 1.467, daytime-plateau flight is 1.315, and daytime-plain flight is 1.196.

The average and median answer to the question “If you only fly daytime-plain flights, what is the maximum time-of-flight in a month that you can fly without fatigue?” are 72.66 and 76 h. The average and median answer to the question “How many hours a month would cause severe fatigue if you only fly daytime-plain flights” are 89.53 and 90 h. According to the average value, if only fly daytime-plain flights in a month, pilots will not be fatigued with time-of-flight less than 73 h, pilots will be more severely fatigued with time-of-flight more than 90 h, and pilots will be mildly fatigued with time-of-flight between 73 and 90 h. From this, the critical value of fatigue coefficient for mild fatigue and severe fatigue is 1.23.

### Model Building

The numerator of Equation 3 is the time-of-flight of each type of flight and the corresponding fatigue correction coefficient, the denominator is the critical fatigue hour when flying daytime-plain flights per month. Using multiple linear regression, the corresponding fatigue feeling value of daytime-plain flight is 1.196. while airlines generally believe that the fatigue correction coefficient of daytime-plain flight is 1, so it is necessary to divide the fatigue feeling value of each type of flight derived from linear regression by 1.196 to obtain the improved fatigue coefficient model.


(3) $$F=\frac{T_{1}\times 1.15+T_{2}\times 1.13+T_{3}\times 1.25+T_{4}\times 1.23+T_{5}\times 1.10+T_{6}}{73}$$

Where *F* is the pilot fatigue coefficient, *T*_1_ is the time-of-flight of morning-plain flight, *T*_2_ is the time-of-flight of evening-plain flight, *T*_3_ is the time-of-flight of morning-plateau flight, *T*_4_ is the time-of-flight of evening-plateau flight, *T*_5_ is the time-of-flight of daytime-plateau flight, *T*_6_ is the time-of-flight of daytime-plain flight. Following the regulations of CAAC Southwest regional administration, it is defined as no fatigue when *F* is less than 1, mild fatigue when *F* is between 1 (included) and 1.23 (not included), and severe fatigue when *F* is greater than 1.23 (included).

### Fatigue Factor Model Effect Evaluation

The data collected include: monthly time-of-flight, time-of-flight of morning flight, time-of-flight of evening flight, time-of-flight of plateau flight, percentage of morning flights among plateau flights, and percentage of daytime flights among plateau flights. After careful examination of the questionnaire data, 599 valid questionnaires of pilot fatigue feeling questionnaires are taken as the overall sample of this model effect evaluation. The minimum monthly time-of-flight was 20 h, the maximum was 98 h.

The collated data were input into the Equations 3 and 4 to obtain the fatigue coefficient values. The fatigue coefficient values were analyzed for consistency between the fatigue level reflected by the fatigue coefficient values and the fatigue level subjectively reflected by the pilots in the questionnaire. In the original model, 288 out of 599 questionnaires were consistent with the fatigue level they answered, accounting for 48.1%, in the new model, 388 out of 599 questionnaires were consistent, accounting for 64.8%.


(4) $$F=\frac{T\times H}{64\,N}$$

Where *T* is the monthly time-of-flight, *H* is the monthly time-of-flight of plateau flight, and *N* is the number of available units.

It can be seen that the percentage of pilots fatigue level that is consistent with their own feelings calculated by the improved new model is significantly higher than that of the original model, which can better reflect the pilots’ fatigue feeling. Through the empirical analysis, it was proved that the new model was better than the original model in terms of accuracy in judging the fatigue level of individual pilots.

## Conclusion

When the current situation of pilot fatigue was analyzed by means of the fatigue scale questionnaire, it was found that the percentage of survey respondents with mild fatigue and above reached 67.7%. In order to further quantify the differences in pilot fatigue caused by different types of flights, this paper divides flights into six categories based on the duty time and whether plateau airports are involved, including daytime-plain flights, morning-plain flights, evening-plain flights, daytime-plateau flights, morning-plateau flights, and evening-plateau flights. Based on the results of the fatigue feeling questionnaire, it was found that the fatigue feeling of flying morning flights was greater than that of flying evening flights, the fatigue feeling of flying evening flights was greater than that of flying daytime flights, and the fatigue feeling of flying plateau flights was greater than that of flying plain flights. The existing pilot fatigue coefficient model was improved by calculating the fatigue feeling values and critical fatigue hours for different types of flights. The empirical analysis found that the accuracy of the new model in determining the fatigue level of individual pilots was improved by 16.7% compared with the original model.

In order to reduce or even avoid flight accidents caused by pilots’ fatigue, the management should impose stricter and more scientific limits on flight time and duty periods. At the same time, the fatigue coefficient, an important indicator, should be added to the company’s fatigue risk management system, so as to alleviate pilot fatigue to a certain extent. Since pilots’ preferences for different flights vary to some extent, airlines should take into account their own conditions and preferences as much as possible when scheduling flights within the regulatory requirements. Moreover, they should strengthen the aero-medical examination of pilots on plateau flights and pay attention to humanistic care, such as improving the sleeping environment of pilots during overnight stays at plateau airports and carrying out psychological counseling work appropriately.

## Data Availability Statement

The data analyzed in this study is subject to the following licenses/restrictions: The date are not publicly available due to privacy or ethical restrictions. Requests to access these datasets should be directed to YW, wangyu@cafuc.edu.cn.

## Author Contributions

PZ: conceptualization and software. YW: data curation. LS: formal analysis and investigation. WZ: methodology and writing — original draft. ZS: resources. HS, WZ, and YW: validation. All authors contributed to the article and approved the submitted version.

## Conflict of Interest

The authors declare that the research was conducted in the absence of any commercial or financial relationships that could be construed as a potential conflict of interest.

## Publisher’s Note

All claims expressed in this article are solely those of the authors and do not necessarily represent those of their affiliated organizations, or those of the publisher, the editors and the reviewers. Any product that may be evaluated in this article, or claim that may be made by its manufacturer, is not guaranteed or endorsed by the publisher.
